# Supplementary material for: Caveolar disruption causes contraction of rat femoral arteries via reduced basal NO release and subsequent closure of BKCa channels
Source: PeerJ. 2015 May 26;3:e966. doi: 10.7717/peerj.966 (PMC4451037; doi:10.7717/peerj.966)
Supplement: Figure S1 [file peerj-03-966-s001.docx]

**Supplementary Information**

Supplementary Figure 1

**Figure S1. Effect of L-NAME on phenylephrine-induced femoral artery contraction.**  **A**. Traces show phenylephrine contractions (0.1 - 30 μM, additions indicated by arrows) before and after incubation with L-NAME (250 µM) **B**. Phenylephrine concentration-response curves before (●) and after (○) incubation with L-NAME. *n*=6.
